# Self-Selection of Interesting Occupation Facilitates Cognitive Response to the Task: An Event-Related Potential Study

**DOI:** 10.3389/fnhum.2020.00299

**Published:** 2020-08-04

**Authors:** Keiichiro Tokuda, Michio Maruta, Suguru Shimokihara, Gwanghee Han, Kounosuke Tomori, Takayuki Tabira

**Affiliations:** ^1^Department of Rehabilitation, Medical Corporation, Gyokusyoukai Takada Hospital, Kagoshima, Japan; ^2^Doctoral Program of Clinical Neuropsychiatry, Graduate School of Health Science, Kagoshima University, Kagoshima, Japan; ^3^Department of Rehabilitation, Medical Corporation, Sansyukai, Okatsu Hospital, Kagoshima, Japan; ^4^Department of Rehabilitation, Medical Corporation, Nissyoukai Minamikagoshimasakura Hospital, Kagoshima, Japan; ^5^Department of Neuropsychiatry, Kumamoto University Hospital, Kumamoto, Japan; ^6^Department of Occupational Therapy, School of Health Science, Tokyo University of Technology, Nishikamata, Ota-Ku, Japan; ^7^Department of Occupational Therapy, School of Health Sciences, Faculty of Medicine, Kagoshima University, Kagoshima, Japan

**Keywords:** self-selection, ADOC, attention, occupational therapy, ERP

## Abstract

**Introduction**: In this study, we examined whether the self-selection of occupations of interest affects reaction times (RTs) and cognitive processing by using the Aid for Decision-making in Occupation Choice (ADOC) and event-related potentials (ERP). We also assessed the relationship of these with psychological indicators.

**Method**: We extracted 78 occupations from the ADOC in consideration of the subjects’ age, and three conditions were set: (1) self-selection of an interesting occupation; (2) self-selection of a disliked occupation; and (3) forced selection. The RT task was executed under their conditions during which ERP was measured. We compared the P300 component of ERP in these conditions. Moreover, we examined the association of cognitive processing and degree of satisfaction and performance concerning occupation, with psychological indicators.

**Results**: P300 amplitude at Fz significantly increased in the self-selection of an interesting occupation. P300 amplitude at Pz was significantly positively correlated with the occupational satisfaction score.

**Conclusion**: Self-selection of interesting occupations in the ADOC resulted in increased attention resource allocation by increasing motivation. Further, there was a positive correlation between satisfaction concerning the occupation and attention of resource allocation. Therefore, occupational therapists should know which occupations the patients consider interesting and help them to select by themselves, thus enhancing their satisfaction after consultation. These interventions may contribute to promoting motivation and cognitive processing.

## Introduction

In recent years, a top-down approach grounded on occupation-based practice has been promoted in the clinical setting of occupational therapy (Nielsen et al., 2017; Nagayama et al., 2018; Maruta et al., 2020). The goal setting is considered a key component of these approaches, with the understanding that selected goals will drive the clinical decision-making process (Levack and Dean, 2012). The Aid for Decision-making in Occupation Choice (ADOC) is a tool to promote the implementation of shared decision-making in occupation-based goal setting (Tomori et al., 2012). Therapists adapt meaningful occupations to rehabilitations in consideration of the degree of satisfaction and performance for occupations after therapists and patients select them by using ADOC. The ADOC makes it possible to share decision making between therapists and patients in an occupation-based goal setting (Tomori et al., 2012, 2015). In this way, it is important for the therapist to share the goals with the patient and to help the patient select the occupations with intention. Moreover, motivational involvement in the occupations is essential for the self-selection of them.

The Self-determination theory provides a comprehensive framework for assessing motivation. Ryan and Deci (2000) classified motivation into three types: no motivation, extrinsic motivation, and intrinsic motivation. It has been reported that intrinsic motivation improves performance and sustainability more than extrinsic motivation (Patall et al., 2008; Areepattamannil et al., 2011). These motivations are positioned on a continuum according to the degree of self-determination. Extrinsic motivation approaches intrinsic motivation by internalizing the external environment and values and integrating them into the self (Ryan and Deci, 2000).

Brain networks have been identified in neuropsychological research on self-selection. Murayama et al. (2015) examined brain occupations using fMRI and compared self-determination (subjects selected the design of a stopwatch by themselves) with forced-determinations (the examiner made the selection). That study revealed that the ventral striatum and medial prefrontal cortex play important roles in the performance of self-selection (Murayama et al., 2015).

On the other hand, event-related potentials (ERP) represent the means to assess cognitive processing with a high temporal resolution (Helfrich and Knight, 2019). The P300 component, which is generated around 300 ms after stimulation in one of the ERP waveforms is triggered by tasks that require cognition and judgment. P300 latency reflects cognitive processing time (Kutas et al., 1977), and the amplitude reflects attention resource allocation (Schubert et al., 1998). Therefore, the study of ERP and P300 components in self-determination and motivation could contribute to the understanding of the cognitive processing and brain activities involved in the performance of self-selection. P300 amplitude was significantly higher in the most motivated participants than in the least motivated ones (Kleih et al., 2010). Further, using ERP, Tanaka et al. (2014) examined cognitive processing responses related to differences in preference. P300 amplitudes for the favorite and disliked animal pictures tended to increase more than the pictures were neither liked nor disliked. In short, P300 amplitudes might promote cognitive processing responses to likes and dislikes (Tanaka et al., 2014).

However, the findings from these studies are difficult to apply in clinical occupational therapy, in terms of meaningful occupations, because of fundamental studies on self-determination and simply comparing differences in preference. We hypothesized that the examination of the P300 component would be responded to during the selection of meaningful and interesting occupations using the ADOC. The result of cognitive processing responses may contribute to emphasizing the importance of goal setting in occupation-based practice.

Therefore, in this study, healthy subjects were asked to select meaningful or disliked occupations using ADOC, and ERP and reaction time (RT) were measured when the occupation images were presented. The aims of this study were: (1) to clarify whether self-selection of both meaningful and interesting occupations increased the cognitive processing of the tasks; and (2) to investigate the association of cognitive processing, degree of satisfaction, and performance with occupation and psychological indicators.

## Materials and Methods

### Subjects

We put up a poster with details of this study on the bulletin board in the Kagoshima University, recruited the participants of the experiment, and used only the applicants as subjects. Twenty-three healthy subjects from Kagoshima University (mean age = 24.1, SD = 5.1, 11 males) participated in this study. All subjects had a normal or corrected-to-normal vision. None of them had a history of neurological or psychiatric disorders or took psychiatric medicine. They gave verbal and written informed consent to participate in the study, but they were not told the aim of the experiments to avoid the effect of information and intended bias on all data. This study was approved by the Ethics Committee on Epidemiological Studies, Kagoshima University (Ref No. 180157).

### Assignments and Stimuli

We used a visual response task and extracted 78 types of occupations from all the 95 tasks of ADOC in consideration of the subjects’ age. We presented visual stimuli and measured RT using an image stimulation system (Multi trigger system MTS0410, Medical Try System, Tokyo). The visual stimuli were presented for 500 ms each, with an interstimulus interval of 2,000 ms, a comprised target stimulus of 30%, and a non-target stimulus of 70%, following a visual oddball paradigm (Maruta et al., 2019). Each condition was finished after the target stimuli were presented 35 times.

### Recording and Data Analysis

Electroencephalogram and evoked potentials, Neuro Pack X1 (NIHON KOHDEN, Tokyo) were monitored using an electromyography tester. EEG was recorded from three scalp sites (Fz, Cz, Pz) according to the 10-20 systems and the sampling rate was 1,000 Hz (Maruta et al., 2019). Ag/AgCl electrodes were referenced to the earlobes and electrode impedance was kept below 5 kΩ. Eye movements were monitored using electrooculograms (EOGs) recorded from electrodes lateral to and below the left eye. For all ERPs, a bandpass filter was applied between 0.5–50 Hz. Remaining epochs were visually inspected, manually removing those containing blinks, eye movements, or other sources of transient noise from the analysis. The peak latency of the P300 components was measured at 250–500 ms. P300 amplitude was defined as the difference in μV from the baseline before the presentation of visual stimuli to the most positive trough between 250–500 ms. When measuring the peak amplitude and latency of the P300 component, some participants showed double peaks of P300. In this case, we selected the largest waveform peak. We also recorded horizontal and vertical electrooculograms to remove artifacts. The P300 component was detected after the ERP waveforms corresponding to the target stimuli were averaged.

### Experimental Protocol

**Figure 1:**
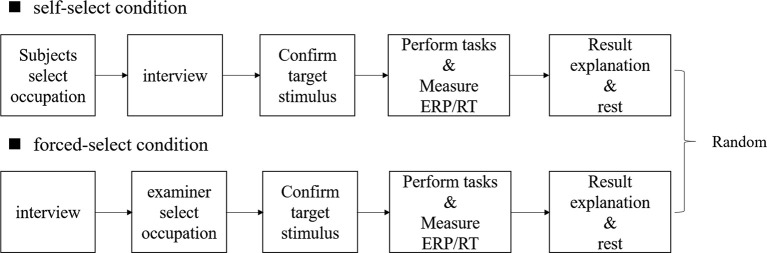
Experimental procedure of the self-selection and the forced-selection conditions. The order of the conditions was randomized, and a 3-min break was provided between each condition.

**Figure 2:**
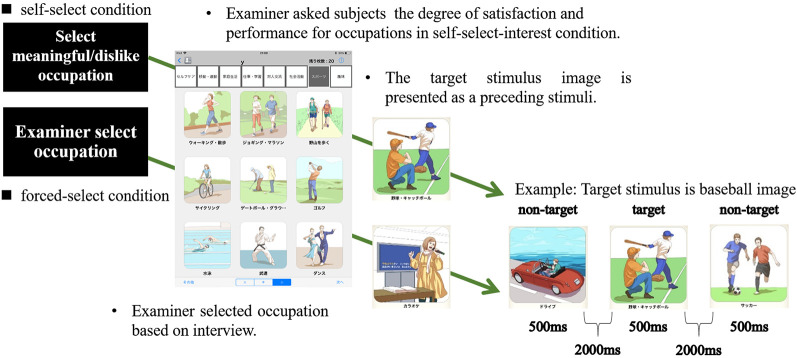
Experimental procedure of the self-selection and forced-selection conditions. Subjects selected the target stimuli in the self-selection condition. Only in the self-selection interesting condition, subjects were asked to indicate their degree of satisfaction and performance for the occupation. We selected the target stimulus based on the interview in the forced-select condition. We conducted visual reaction tasks after the target stimuli were confirmed.

There were three experimental conditions for different visual stimuli. In the first condition, the subjects were asked to select an occupation they considered both meaningful and interesting from a list of 78 occupations using ADOC (self-selection meaningful condition: SSMC). In the second condition, they were asked to select an occupation they disliked (self-selection dislike condition: SSDC). In the third condition, we selected an occupation that was neither liked nor disliked for the subjects (forced-select condition: FSC). The three conditions were performed in random order. The subjects were introduced with the experimental method and asked to practice 10 times before starting the experiment. Subjects were instructed to push a handgrip button to target stimuli with their dominant thumb as accurately and quickly as possible while seated approximately 100 cm from a 19-inch LCD monitor, and ERP was measured during the tasks. Each condition was finished after the target stimuli were presented 35 times. A 3 min rest was provided between conditions (Figure 1). In the SSMC, we examined satisfaction and performance scores for self-selection of meaningful occupation. The participants were asked to provide a satisfaction score for their meaningful and interesting occupations ranging from 1, extremely unsatisfied, to 10, extremely satisfied. In the same way, they were asked to provide a performance score. A 10-point score meant that the participants were good at the selected occupation, and 1 point meant that they could not do it at all (Figure 2). Subjects were instructed to push the handgrip button to target the stimuli with their dominant thumb as accurately and quickly as possible, while seated approximately 100 cm from a 19-inch LCD monitor and viewing the ADOC images.

### Psychological Indicators

The Japanese version of the Maudsley Personality Inventory (JMPI) was administered to the subjects to examine the association between introverted/extroverted personality types (E score) and cognitive processing. The JMPI consists of extroversion-introversion (E scale, 24 items), the neurotic tendency (N scale, 24 items), a false scale (L scale, 20 items), and 12 neutral items (Iwasaki et al., 1970). High scores are proportional to extroversion on the E scale (Arthur and Jensen, 1958).

### Statistical Analysis

We conducted a Shapiro–Wilk test to test the normality of the P300 component among the three conditions, and normality was confirmed. We used repeated-measures-ANOVA to compare P300 amplitude, latency, and RT in the three conditions, followed by Bonferroni’s *post hoc* comparisons at *P* < 0.05 of significance. Moreover, we used Spearman’s rank correlation coefficient to investigate the association between P300 components as the dependent variable, and satisfaction/performance scores for occupation, E score as independent variables. Statistical analyses were performed with SPSS version 25.0.

## Result

### Summary of the Subjects of the Study

**Figure 3:**
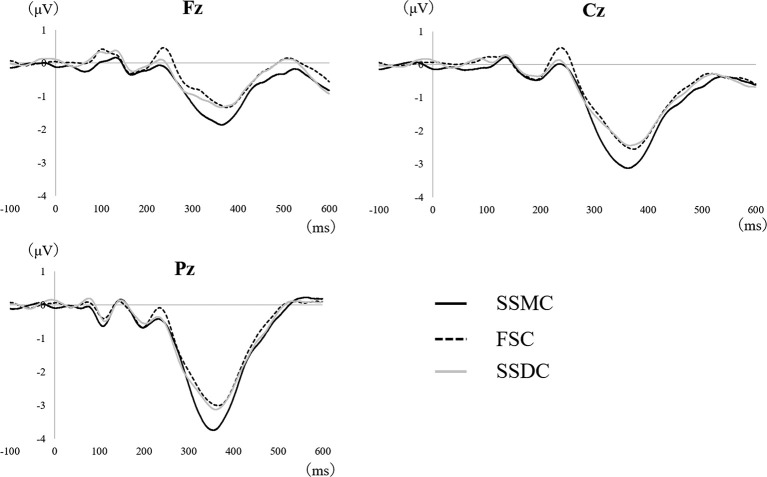
Event-related potentials (ERP) averaging waveforms were obtained between the self-selection and forced-selection conditions (*N* = 22). The average waveforms of ERP obtained in all conditions were shown for three scalp sites. FSC, forced-selection condition; SSDC, self-selection dislike condition; SSMC, self-selection meaningful condition.

We investigated 22 subjects (mean age = 24.3, SD = 5.2, 11 males) because one subject had less than 20 waveforms without artifacts in each condition (Cohen and Polish, 1997). Figure 3 shows the P300 average waveform obtained under each condition. All the errors (two subjects each made an error twice) during task performance were generated only when non-target visual stimuli were presented, so the results were not affected.

### P300 Component and RT

**Figure 4:**
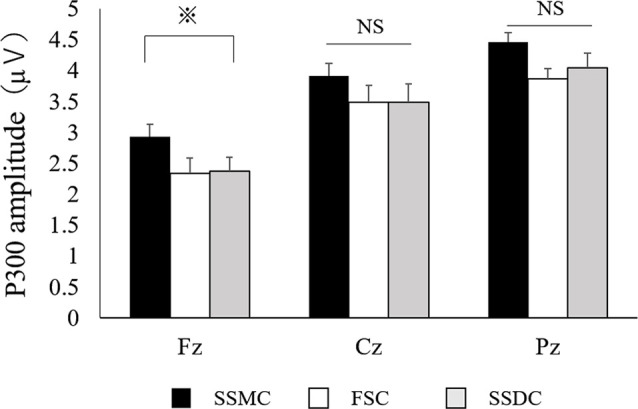
Comparison of P300 amplitude between the self-selection and forced-selection conditions. The results of the P300 amplitude were analyzed using repeated-measures-ANOVA followed by Bonferroni *post hoc* tests. P300 amplitude at Fz significantly increased more in SSMC. FSC, forced-selection condition; SSDC, self-selection dislike condition; SSMC, self-select-meaningful condition; NS, not significant; ^⋇^*P* < 0.05.

**Figure 5:**
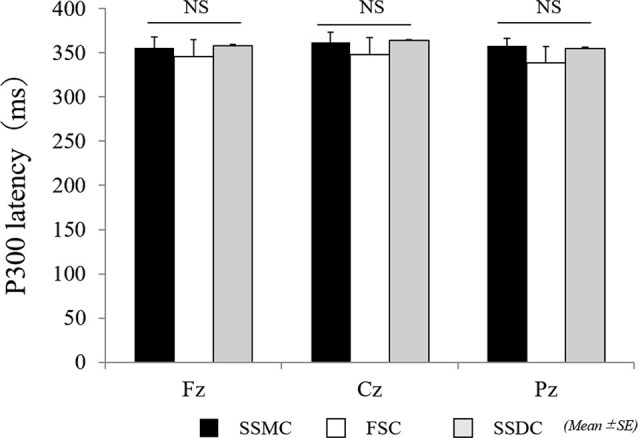
Comparison of P300 latency between self-select and forced-select condition. The results of P300 latency were analyzed using repeated-measures-ANOVA. No significant effect was found. FSC, forced-selection condition; SSDC, self-selection dislike condition; SSMC, self-selection meaningful condition; NS, not significant.

**Figure 6:**
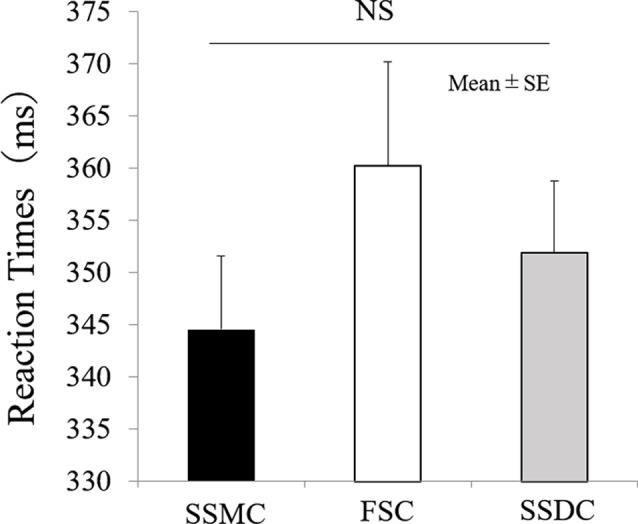
Comparison of RT between self-select and forced-select condition. The results of RT were shown in repeated-measures-ANOVA. No main effect was found. FSC, forced-selection condition; RT, reaction time; SSDC, self-selection dislike condition; SSMC, self-selection meaningful condition; NS, not significant.

The main difference between the three conditions was observed in the P300 amplitude at Fz (*F*_(2,42)_ = 6.2, *P* = 0.004). After examining each condition, the P300 amplitude at Fz (*F*_(2,42)_ = 6.2, *P* = 0.004) in the SSMC was more significantly increased than in the other two conditions (SSMC vs. SSDC; *P* = 0.02, SSMC vs. FSC; *P* = 0.04). There was no difference between SSDC and FSC (Figure 4). Moreover, there was no significant difference at Cz (*F*_(2,42)_ = 2.1, *P* = 0.13) or Pz (*F*_(2,42)_ = 3.1, *P* = 0.06). The difference in P300 latency and RT between the conditions was not significant (Figures 5, 6).

### Association Between P300 Component, RT, Satisfaction, Performance, and Psychological Index in the SSMC

**Figure 7:**
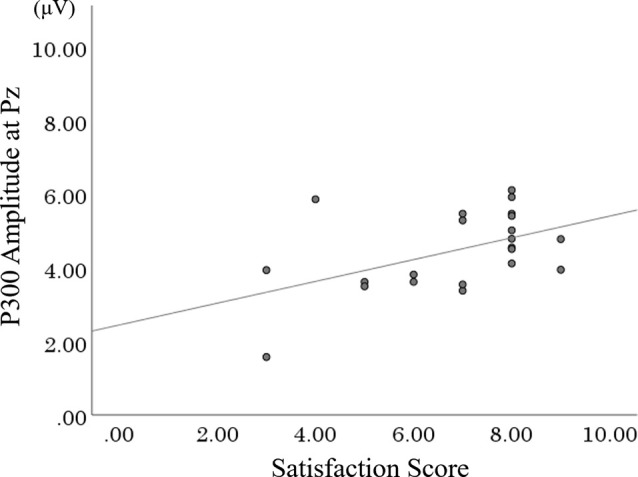
Scatter plot of the correlation between P300 amplitude at Pz and satisfaction score in SSMC. SSMC, self-selection meaningful condition.

P300 amplitude at Pz was significantly and positively correlated with occupational satisfaction score (Figure 7). The high score of the performance degree did not significantly increase. No significant correlation between the P300 component and extroversion-introversion (Table 1).

**Table 1:** Association between P300 component, RT, satisfaction, performance, and E score in the self-selection interesting condition.

	SSMC P300 Amp			SSMC P300 Lat			RT
	Fz	Cz	Pz	Fz	Cz	Pz
Satisfaction	0.371	0.418	0.457*	0.23	0.242	0.054	0.382
Performance	0.414	0.325	0.368	−0.026	−0.038	−0.029	0.235
E score	−0.274	−0.392	−0.381	0.292	0.152	0.261	−0.063

SSMC, self-selection meaningful condition. Amp, Amplitude; Lat, Latency; RT, Reaction Time; E score, introverted/extroverted personalities types (JMPI); Spearman’s rank correlation coefficient, Correlation coefficient, *P < 0.05.

## Discussion

### Self-Selection Interesting Condition and P300 Amplitude

We found that the P300 amplitude at Fz in SSMC was more significantly increased than in SSDC and FSC.

P300 amplitude reflects attention resource allocation to the task (Kaga et al., 1995) and is caused by factors such as task relevance, motivation, and alertness (Olofsson et al., 2008). P300 amplitude in the self-determination condition resulted in a more significant increase than in the forced-determination condition when the oddball task was conducted by using character images as visual stimuli (Maruta et al., 2019). Further, Suzuki et al. (2005) reported that P300 amplitude was significantly decreased while watching an interesting video than uninteresting videos in auditory stimuli task. Therefore, selecting an interesting and meaningful occupation in the ADOC suggests that attention resource allocation to the presented stimuli more increases than others’ choices (SSDC and FSC).

The P300 source has not been accurately revealed various investigations on the subject. Mulert et al. (2004) suggested that the P300 source is involved in areas such as the inferior parietal lobule, temporal-parietal junction, supplementary motor cortex, anterior cingulate cortex, and superior temporal gyrus. Especially, it has been reported that the dorsal anterior cingulate cortex is activated, requiring continuous adjustment of attention distribution (Tops and Boskem, 2011). Moreover, the dorsal anterior cingulate cortex, supplementary motor area, and front insula were significantly more activated in the self-determination condition (subjects selected the design of the task tools) than in the forced-determination condition (in which the examiner made the selection; Murayama et al., 2015). These previous studies could suggest that the anterior cingulate cortex is part of the P300 origin and is activated during self-determination.

In this study, P300 amplitude might have increased significantly in SSMC because of the self-selection of interesting occupations enhances motivation and facilitates the allocation of attention resources to target stimuli. Moreover, the P300 amplitude at Fz increased, which was consistent with the results of Maruta et al. (2019). It is possible that the anterior cingulate cortex, which is activated in the SSMC, reflected P300 amplitude in this study. However, this hypothesis cannot be readily accepted because we did not analyze brain function images.

### Self-Selection Interesting Condition and P300 Latency and RT

We found that P300 latency and RT were not different between the conditions. According to previous studies, they are related to the perception processing time of stimuli (Kutas et al., 1977; Duncan-Johnson, 1981), and depend on age and task difficulty (McCarthy and Donchin, 1981; Takakura et al., 2016).

The results of the present study suggest that self-selection of interesting occupations had no significant effect on perceptual processing time because there is no significant difference in subject’s age between groups and the same stimuli were presented in all conditions.

### Association Between the Degree of Satisfaction for Interesting Occupations and P300

In the present study, the degree of satisfaction for the selected interesting occupation and the P300 amplitude at Pz showed a moderate positive correlation in the SSMC.

Meaningful activity (interesting occupation) affects the fulfillment of basic psychological needs (autonomy, relevance, and ability; Eakman, 2014). These are the basic needs on which self-determination theory is based. Meaningful occupations help to motivate by satisfying these needs. Moreover, the degree of satisfaction with daily occupations is relative to the self-evaluation of quality of life (Eklund and Leufstadius, 2007).

In this study, the self-selection of interesting and meaningful occupations increased motivation by fulfilling basic psychological needs, and their satisfaction could have promoted more cognitive processing than the other conditions (SSDC and FSC). In clinical practice, setting a goal that can enhance the satisfaction of a meaningful occupation may contribute to promoting cognitive processing responses, increasing motivation, and improving quality of life.

### Association Between Psychological Characteristics and P300

The analysis of examining the association between the E score by the Maudsley personality test and the P300 component showed no association between the degree of satisfaction and performance for occupation and E score in the SSMC.

Cahill and Polish (1992) reported that extroverts tended to show a higher P300 amplitude than introverts did in an oddball task.

In contrast with the latter study, we used the participants’ meaningful occupations as target stimuli instead of ranges, and so the stimuli properties and instructions were fundamentally different. Therefore, due to the differences in the methods used, our results from the experiments using visual stimuli suggest that P300 and psychological characteristics were not related.

### Limitations and Issues of This Research

Regarding the limitations and issues of this research, the following points can be mentioned. The subjects of this study were young people, therefore it is necessary to investigate this phenomenon in a wide range of ages and clinical situations. Also, the ERP results in this study revealed only a tendency, and analysis of brain function images was not performed. This study was preliminarily conducted with only three basic brain sites as the first step to a bridge-setting goal and basic research in occupational therapy. Further research needs to explore the association between self-selection of interesting occupations and brain function.

## Conclusion

The selection of an interesting and meaningful occupation in the ADOC promotes cognitive processing by increasing motivation and attention resource allocation. Moreover, since we found a moderate correlation between the degree of satisfaction with occupation and attention resource allocation, we suggest that the degree of satisfaction reported for the occupations affects cognitive processing. Occupational therapists should know which occupations the patient considers interesting, and help them to select one by themselves, and, thus, may be enhancing their satisfaction after consultation. These interventions may contribute to promoting motivation and cognitive processing. Our main conclusion is that selecting an interesting and meaningful occupation promotes cognitive processing.

## Data Availability Statement

All datasets presented in this study are included in the article.

## Ethics Statement

The studies involving human participants were reviewed and approved by Ethics Committee on Epidemiological Studies, Kagoshima University. The patients/participants provided their written informed consent to participate in this study.

## Author Contributions

KTok conceived the concept of the manuscript. KTok wrote the first draft of the manuscript. MM, SS, GH, KTom and TT wrote the sections of the draft. All authors contributed to the article and approved the submitted version.

## Conflict of Interest

KTok was employed by the company Department of Rehabilitation, Medical Corporation, Gyokusyoukai Takada Hospital. The remaining authors declare that the research was conducted in the absence of any commercial or financial relationships that could be construed as a potential conflict of interest.
